# A novel potential mechanism for the development of portal vein thrombosis in cirrhosis based on portal hemodynamics

**DOI:** 10.1186/s13244-022-01330-4

**Published:** 2022-12-13

**Authors:** Yuling Yan, Zhuxiang Xiong, Xiaoze Wang, Li Yang, Tinghui Zheng, Xuefeng Luo

**Affiliations:** 1 Department of Gastroenterology and Hepatology, West China Hospital, Sichuan University, 37 Guoxue Lane, Chengdu, 610041 Sichuan People’s Republic of China; 2 Sichuan University-University of Oxford Huaxi Joint for Gastrointestinal Cancer Centre, Chengdu, Sichuan People’s Republic of China; 3 Department of Applied Mechanics, Sichuan University. No, 24 South Section of First Ring Road,, Chengdu, 610065 Sichuan Province People’s Republic of China; 4 Yibin Institute of Industrial Technology/Sichuan University Yibin Park, Yibin, People’s Republic of China

**Keywords:** Portal vein thrombosis, Liver cirrhosis, Helical flow, Localized normalized helicity, Helicity intensity

## Abstract

**Background:**

Marked changes in hemodynamics have been suggested to be a potential contributing factor to portal vein thrombosis (PVT) development. This study investigated the effect of portal hemodynamics based on the anatomical structure of the portal venous system on PVT development.

**Methods:**

The morphological features of portal venous system in patients with PVT and those without PVT subgroups were compared. In addition, idealized PV models were established to numerically evaluate the effect of the variation in the angulation of superior mesenteric vein (SMV) and splenic vein (SV) on the hemodynamics of portal venous system.

**Results:**

The angle α (angulation of SMV and SV) in patients with PVT was lower than that in patients without PVT (*p* < 0.0001), which was the only independent risk factor (odds ratio (OR), 0.90 (95% CI 0.84–0.95); *p* < 0.0001) for the presence of PVT. With the change in angle α, the flow pattern of blood flow changed greatly, especially the helical flow. When α = 80°, helical flow only appeared at the local PV near the intersection of SMV and SV. When α = 120°, most regions were occupied by the helical flow. In addition, the h_2_ gradually increased with increasing α, when α = 80°, *h*_2_ = 12.6 m/s^2^; when *α* = 120°, *h*_2_ = 29.3 m/s^2^.

**Conclusions:**

The angulation of SV and SMV was closely associated with PVT development. Helical flow changed following the varying angulation of SV and SMV. Therefore, angulation of SV and SMV may help to identify high-risk cohorts for future PVT development earlier.

**Supplementary Information:**

The online version contains supplementary material available at 10.1186/s13244-022-01330-4.

## Key points

1. The angulation of SMV and SV in patients with PVT was lower than that in patients without PVT.
2. With the change in angulation of the SMV and SV, the flow pattern of blood flow changed greatly, especially the helical flow, which is associated with the presence of PVT.
3. The change in portal hemodynamics is associated with the development of PVT.

**Supplementary Information:**

The online version contains supplementary material available at 10.1186/s13244-022-01330-4.

## Background

Portal vein thrombosis (PVT) is a common complication in patients with cirrhosis, and its prevalence in liver cirrhosis ranges from 2.1 to 16.2%, which is higher in patients waiting for liver transplantation, ranging from 5.5 to 26% [1]. Numerous studies have found that PVT is associated with worse outcomes for pre-and post-liver transplantation [2–4]. Moreover, PVT may exacerbate portal hypertension and contribute to portal hypertension-related variceal bleeding, thus could possibly increasing the risk for the development of acute decompensation [5].

PVT development in patients with cirrhosis is multifactorial. Patients with cirrhosis have a well-described derangement of the hemostatic balance due to a reduction in both anticoagulant and procoagulant factors [6]. In addition, worse liver function (Child–Pugh class B and C), nonselective beta-blockers (NSBBs) taking history, endoscopic therapy for esophageal varices and a past history of variceal bleeding are also suggested to play a role in developing PVT [7–9]. However, controversies remain about whether these factors are able to sufficiently account for the clinically observed interpatient differences in the risk of PVT [10].

The marked changes in hemodynamics in cirrhosis have been suggested to be a potential contributing factor to the formation of PVT. When the portal vein (PV) velocity decreases to 15 cm/s, patients with cirrhosis have a highly significant risk association with the future development of PVT [11], and the changes in hemodynamic characteristics after splenectomy increase the risk of PVT development [12, 13]. While the anatomical structure of the portal venous system plays a significant role in the development of its flow features and hemodynamic parameters. For instance, the orientation of the inlet vessels is reported to significantly affect the flow distribution in the hepatic venous system [14]. Moreover, the helicity of the helical flow in the portal vein which might be important to minimize the clinical risks of developing thrombus [15] and it was found to be strongly correlated with the angulation of superior mesenteric vein (SMV) and splenic vein (SV) [16], indicating that the relationship between PV structure and flow may be of clinical importance. In addition, several liver surgical studies found that the changes in angle of portal vein have an effect on PVT development [17, 18]. However, up to now, whether the portal hemodynamics based on the anatomical structure can affect the PVT development in patients with cirrhosis has not yet determined.

In this study, we compared the morphological features of portal venous system in patients with PVT to those without PVT, including the diameter of the main vein and the angulation of the SMV and SV, etc. In addition, idealized PV models were established to numerically evaluate the effect of the variation of the angulation of SMV and SV on the hemodynamics of the portal venous system to investigate the potential risk of PVT.

## Materials and methods

### Study population

This was a retrospective study, and the detailed CTA images of patients preparing for transjugular intrahepatic portosystemic shunt (TIPS) at our hospital between February, 2017, and February, 2018, were collected. All included patients had definite cirrhosis in CT images. Patients with PVT were first collected, and patients without PVT were matched one-to-one. PVT was defined as low-density area within the portal vein in portal vein phase CT images. The inclusion criteria: patients with definite diagnosis of cirrhosis on CT image. Patients with PVT that did not completely occlude the portal vein trunk. The exclusion criteria were as follows: patients with splenic embolization, splenectomy, hepatocellular carcinoma or cavernous transformation of portal vein.

### CTA acquisition and analysis

Thin-slice CTA images of the abdomen were generally obtained using a second-generation dual-source CT scanner (Somatom Definition; Siemens Healthcare, Erlangen, Germany). Abdominal CT angiography is generally performed in 4 phases (plain scan, arterial phase, portal vein phase, and vein phase). The three-dimensional portal vein geometries were then reconstructed from the portal vein phase CT images by the same investigator through a rigorous approach. The commercially available software Mimics (Materialise, Plymouth, Mich) was used for analysis.

### Morphological features

**Fig. 1:**
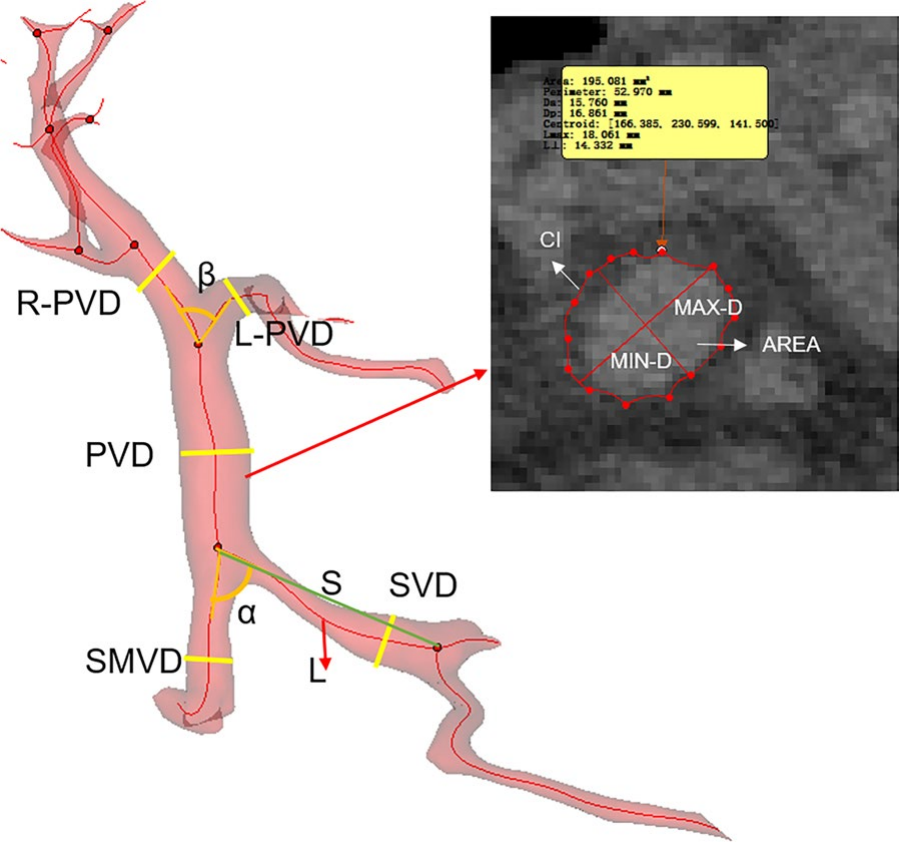
Illustration of the method of measurement of morphological features. PVD, portal vein diameter; L-PVD, left portal vein diameter; R-PVD, right portal vein diameter; SMVD, superior mesenteric vein diameter; SVD, splenic vein diameter; MAX-D, maximum diameter at the PV cross section; MIN-D, minimum diameter at the PV cross section; AREA, the area of PV cross section; CI, Circumference of PV cross section; α, the angle of SMV and SV; β, the angle of LPV and RPV; S, the linear distance from the starting point of the SV to hilus lienis; L, the length of center line of the SV from the starting point of the SV to hilus lienis

The portal vein diameter (PVD), left portal vein diameter (L-PVD), right portal vein diameter (R-PVD), maximum diameter of PV, minimum diameter of PV, length of PV, curvature of PV, area of PV, circumference of PV, splenic vein diameter (SVD), curvature of SV, superior mesenteric vein diameter (SMVD), angulation of SV and SMV (α, in anterior–posterior axis), angle of PV and SV (β, in XY axial plane) and hydraulic diameter of PV were measured. In addition, the curvature (CU) of SV was calculated by the formula CU = (L-S)/L, where L and S were the length of centerline of the SV and the linear distance from the starting point of the SV to hilus lienis, respectively (Fig. 1).

### Hemodynamic parameter simulation

#### *Geometricalmodels*

To analyze the effect of morphological features on portal flow hemodynamics, the ideal model of the portal vein was established using the commercial software SolidWorks. This model includes the main portal vein (PV) and its left (LPV) and right branches (RPV), superior mesenteric vein (SMV), inferior mesenteric vein (IMV), splenic vein (SV) and left gastric vein (LGV) (Additional file 1: Figure S1). Among them, the diameters of LPV and RPV were 7.48 mm and 9.19 mm, respectively. The IMV merges into the SV at a distance of 27.6 mm from the intersection of the SMV and PV, and forms an angle of 70° with the SV; the diameter of the LGV was 4.92 mm, which also merges into the SV. The diameters of the PV trunk and SV were 13 mm and 10 mm, respectively. In addition, the angulation of the SMV and SV was 100°. The inlet and outlets were cut orthogonally to the centerline and extended 10 times the vein diameter to ensure that the boundary conditions would not affect the flow field within the veins. The reconstructed models were imported into ANSYS ICEM for mesh generation. An unstructured mesh that consists of tetrahedral cells combined with prismatic cells near the wall was created. The prism-layer mesh was progressively refined near the wall. For a better discretization of small veins, the thickness of this mesh and surface size (edge length) were defined relative to the local lumen diameter.

#### Governing equation

In the portal system, the pulsation characteristics of blood flow were not significant, so the numerical simulation in this study is simplified to steady simulation. In addition, the blood, as a preliminary study, was assumed to be incompressible, laminar, homogenous and Newtonian. The corresponding governing equations were as follows:

1 $$\rho \left( {\vec{u} \cdot \nabla } \right)\vec{u} + \nabla p - \mu \nabla^{2} \vec{u} = 0$$

2 $$\nabla \cdot \vec{u} = 0$$

where $\vec{u}$ and $p$ are the fluid velocity vector and pressure, respectively, and $\rho$ and $\mu$ are the fluid density of 1050 kg/m^3^ and viscosity of 0.0035 Pa s, respectively.

#### Boundary conditions

To investigate the effect of the angulation of the SMV and SV on the hemodynamics in the portal system, the angulation varied from 80° to 120° and the other parameters were unchanged. The fixed velocities at SMV, SV, LGV and IMV inlets were set to be 13.87 cm/s, 18.30 cm/s, 8.00 cm/s and 7.80 cm/s, respectively [15, 18]. The RPV and LPV outlets were fixed at 10 mmHg, and the vein walls were assumed to be rigid.

#### Numerical simulation

Commercial CFD software (Ansys FLUENT 16.0) was utilized for the numerical simulation. The default segregate implicit solver was applied to all equations, SIMPLE was adopted to couple the outflow velocity terms, and the convergence criterion was set to 1e^−5^.

The model with α equal to 100°) was employed for the mesh independence study. The computational domain was covered with 1.02 million elements and 2.59 million elements. The maximum WSS difference among the models with coarse and fine meshes was less than 5%. In addition, the computational costs (Intel Platinum 8180 2.6G 10.4UPI 19.25 M 14C 140 W) were 3.2 h and 7.8 h with the coarse mesh and fine mesh, respectively. Therefore, a coarse mesh was used for the study.

### Helical flow measurement

Helical flow in the PV was calculated: the localized normalized helicity (LNH) and helicity intensity (*h*_2_), respectively, represent local blood flow, and the absolute h_2_ of the specified computational domain within the rotation direction and period, respectively. The calculation formula of LNH and h_2_ is as follows:

3 $${\text{LNH}}\left( x \right) = \frac{v\left( x \right) \cdot \omega \left( x \right)}{{\left| {v\left( x \right)} \right|\left| {\omega \left( x \right)} \right|}} = \cos \alpha \left( x \right)$$

4 $$H = \mathop \smallint \limits_{D}^{{}} v\left( x \right) \cdot \nabla \times v\left( x \right){\text{d}}V = \mathop \smallint \limits_{D}^{{}} H_{k} \left( x \right){\text{d}}V$$

5 $$h_{2} = \frac{1}{Vi}\mathop \smallint \limits_{Vi}^{{}} \left| {H_{k} } \right|{\text{d}}V$$

where *v*(*x*) and *ω*(*x*) are the velocity and vortex vectors, respectively, and *D* and *V* are the fluid domains.

### Statistical analysis

Data were expressed as mean ± the standard deviation or as median (range) depending on the distribution. The Student’s t test or Mann–Whitney test was used to compare characteristics in patients with PVT and without PVT subgroups. The receiving operating characteristic curve (ROC) analysis was used to evaluate the performance of morphological features for predicting PVT. Characteristics were analyzed with univariate logistic regression analysis, and those with *p* < 0.10 were subsequently included in multivariable logistic regression analysis. SPSS software (version 23.0, SPSS) was applied for calculation. *p* < 0.05 indicated that the difference was statistically significant.

## Results

### Baseline characteristics

A total of 142 patients with CTA were included; among them 48 patients with PVT, 4 patients with splenectomy history, 7 patients with cavernous transformation of the portal vein and 1 patient with splenic embolization history were excluded. Finally, 36 patients with PVT were enrolled. For one-to-one matching, 36 patients without PVT were enrolled.

**Fig. 2:**
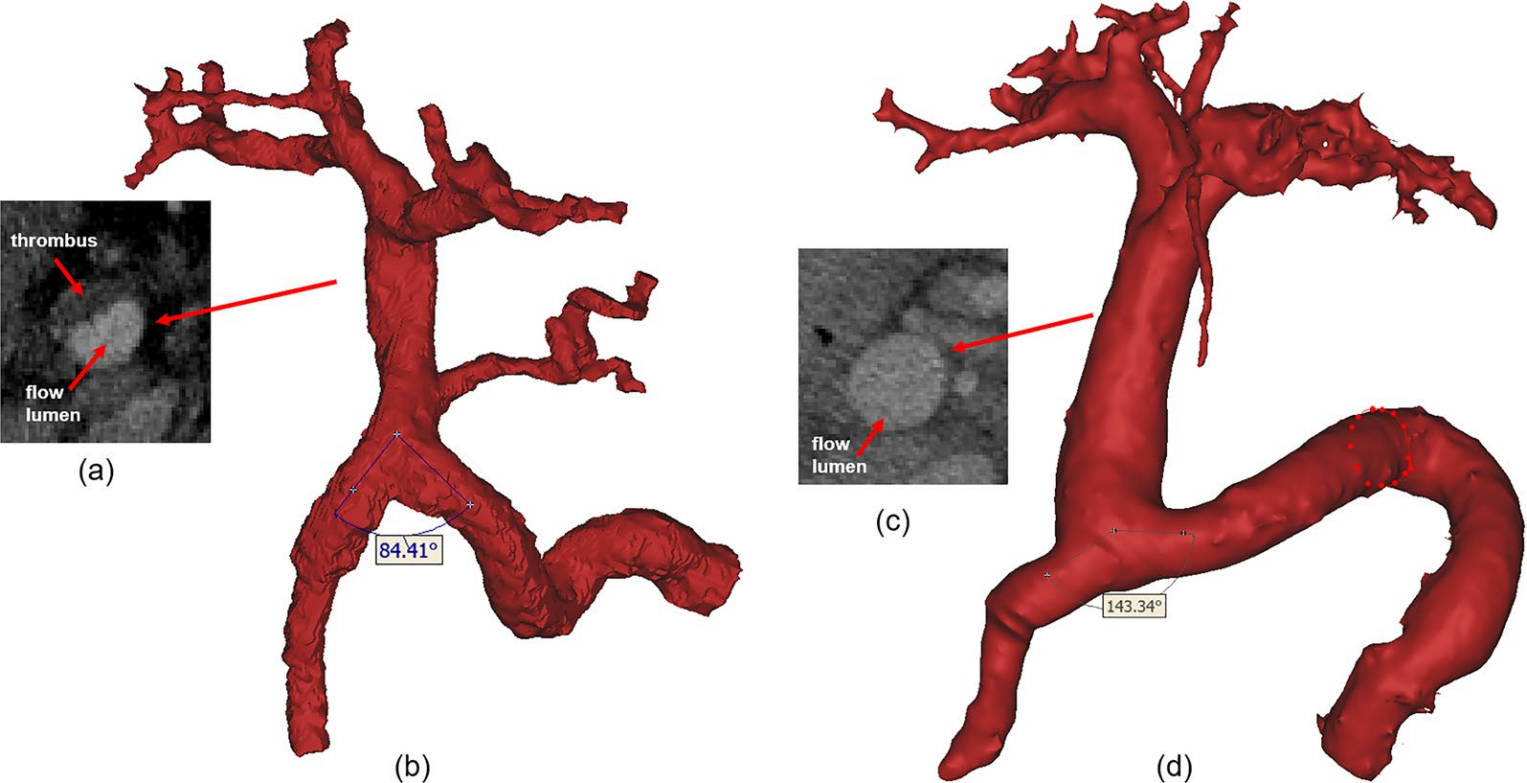
The represented morphology in patients with PVT (**b**) and without PVT (**d**); **a** and **c** show the detail of portal vein cross section in the patient with PVT it without PVT, respectively

The baseline characteristics of patients with PVT and patients without PVT are shown in Table 1. There was nearly no difference between the two groups, only the endoscopic variceal ligation (EVL) and/or endoscopic injection sclerotherapy (EIS) history in patients with PVT was more than that in patients without PVT (*p* = 0.04)*.* The morphological features between the patients with PVT and without PVT groups were compared (Table 2). Among them, the angulation of SMV and SV (*p* < 0.0001), curvature of PV (*p* = 0.003), maximum diameter of PV (*p* = 0.04) and angle of PV (*p* = 0.003) were significantly different between the two groups. The represented morphology in patients with PVT and without PVT is shown in Fig. 2.

**Table 1:** Baseline characteristics of enrolled patients

Characteristic	With PVT *(n* = *36)*	Without PVT *(n* = *36)*
Gender, male (%)	28 (77.8%)	29 (80.6%)
Age, years	51 ± 10	51 ± 11
The etiology		
HBV	20	27
HCV	5	2
Alcohol	8	7
Others	4	0
PLT, × 10^9^ /L	67 (44–96)	64 (40–86)
Bilirubin, μmol /L	19.8 (13.9–27.3)	19.6 (15.2–27.5)
Albumin, g/L	34.2 ± 5.3	34.0 ± 4.6
ALT, IU/L	20 (16–24)	25 (16–40)
AST, IU/L	21 (21–36)	32 (24–53)
Creatinine, μmol /L	67 (56–79)	71 (62–85)
PT, S	15.0 ± 1.9	14.7 ± 1.9
INR	1.3 ± 0.2	1.3 ± 0.2
MELD	10 (9–12)	10 (9–12)
Child–Pugh score		
A	12	7
B	16	22
C	8	7
EVL and/or EIS history	15	8^∗^
Ascites		
Without ascites	7	4
I	12	9
II	3	9
III	14	14
PPG, mmHg	22 ± 6	21 ± 5

Data are presented as median (interquartile range) or number (percentage). PVT, portal vein thrombosis; HBV, hepatitis B virus; HCV, hepatitis B virus; PLT, platelet count; ALT, alanine aminotransferase; AST, aspartate aminotransferase; PT, prothrombin time; INR, international normalized ratio; MELD, model for end-stage liver disease; EVL, endoscopic variceal ligation; EIS, endoscopic injection sclerotherapy; PPG, portal pressure gradient

**Table 2:** Baseline morphological characteristics in patients with PVT and without PVT

Characteristic	With PVT (*n* = 36)	Without PVT (*n* = 36)	*p *value
PV diameter, mm	15.6 ± 3.2	14.9 ± 2.5	0.31
SMV diameter, mm	11.5 ± 2.5	10.7 ± 2.0	0.14
SV diameter, mm	12.1 ± 4.0	11.7 ± 3.3	0.59
Angulation of SV and SMV	97.3 ± 12.8	114.5 ± 14.3	< 0.0001
Curvature of SV	0.3 ± 0.1	0.3 ± 0.1	0.91
LPV diameter, mm	11.0 ± 3.6	10.6 ± 2.6	0.57
RPV diameter, mm	10.7 ± 4.1	11.3 ± 2.7	0.56
Angle of LPV and RPV	95.5 ± 16.4	95.2 ± 19.7	0.94
MAX PV diameter, mm	18.0 ± 5.2	16.0 ± 2.6	0.04
MIN PV diameter, mm	13.6 ± 3.2	13.8 ± 2.4	0.75
PV length, mm	54.5 ± 11.3	58.5 ± 9.5	0.11
Angle of PV	117.5 ± 11.3	126.8 ± 11.4	0.003
Curvature of PV	0.04 (0.03–0.08)	0.03 (0.02–0.04)	0.003
Area of PV	255 (169–330)	216 (170–264)	0.05
Circumference of PV	59.6 ± 13.8	55.4 ± 8.3	0.12
Hydraulic diameter of PV	17.2 ± 4.3	15.7 ± 2.5	0.07

PV, portal vein; SMV, superior mesenteric vein; SV, splenic vein; LPV, left portal vein; RPV, right portal vein; MAX, maximum; MIN, minimum

### The ability of morphological features for diagnosing PVT

**Fig. 3:**
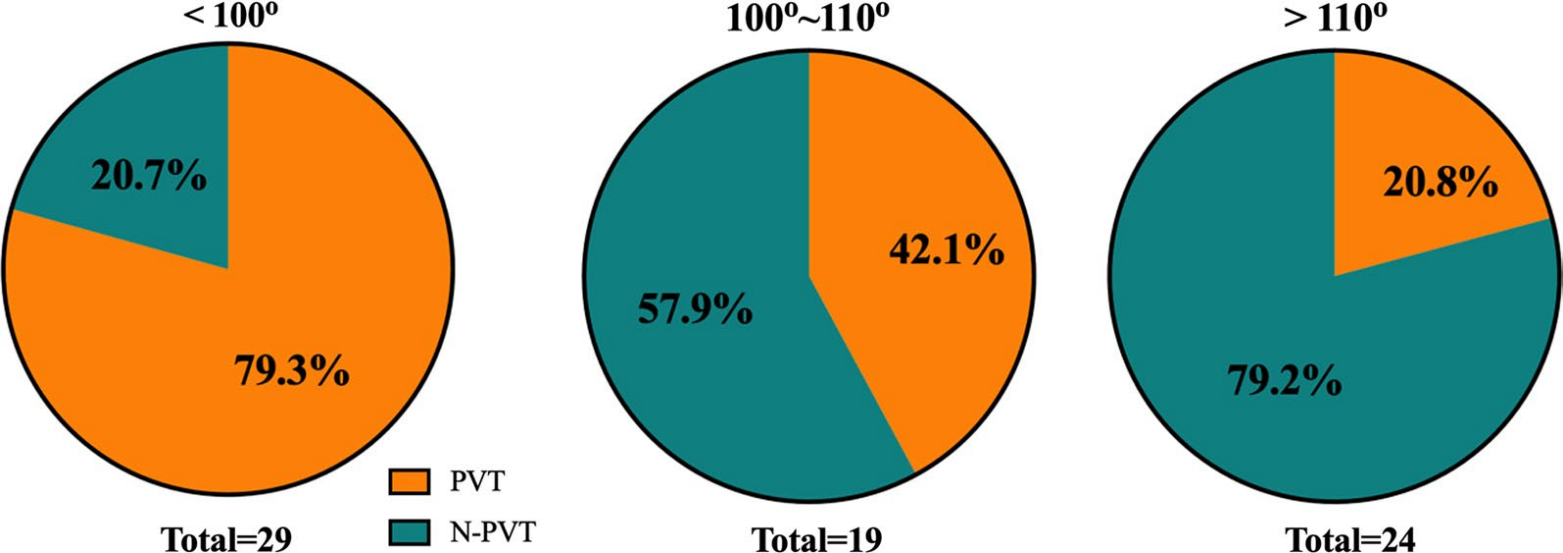
The prevalence of PVT according to degree of angulations of SMV and SV

In univariate logistic regression analysis, angulation of SMV and SV (*p* < 0.0001), maximum diameter of PV (*p* = 0.06), curvature of PV (*p* = 0.007), EVL or EIS history (*p* = 0.08), angle of PV (*p* = 0.005), area of PV (*p* = 0.07) and hydraulic diameter of PV (*p* = 0.07) were associated with the presence of PVT. In the multivariable logistic regression analysis, only angulation of SMV and SV (odds ratio (OR), 0.90 (95% CI 0.84–0.95); *p* < 0.0001) were independent risk factors for the presence of PVT (Table 3). The AUC of angulation of SMV and SV for predicting PVT was 0.81 (95%CI: 0.71–0.91) (Additional file 1: Figure S2). According to the degree of angulations of the SMV and SV, the prevalence of PVT was calculated (Fig. 3). Twenty-nine patients with angulation of SMV and SV < 100°, among them, 23 patients had PVT and 6 patients did not have PVT. Twenty-four patients had angulations of SMV and SV > 110°, among them, 19 patients did not have PVT and 5 patients had PVT. Nineteen patients with angulations of SMV and SV between 100° and 110°; among them, 8 patients had PVT, and 11 patients did not have PVT.

**Table 3:** Univariable and multivariable linear regression analyses of parameters associated with PVT

	Univariate		Multivariable	
	Odds ratio (95% CI)	*p* Value	Odds ratio (95% CI)	*p* Value
PV diameter, mm	1.09 (0.93–1.28)	0.31		
SMV diameter, mm	1.17 (0.95–1.45)	0.14		
SV diameter, mm	1.04 (0.91–1.18)	0.59		
Angulation of SV and SMV	0.91 (0.86–0.95)	< 0.0001	0.90 (0.85–0.95)	< 0.0001
Curvature of SV	0.82 (0.03–25.67)	0.91		
LPV diameter, mm	1.01 (0.88–1.16)	0.87		
RPV diameter, mm	0.93 (0.83–1.04)	0.18		
Angulation of LPV and RPV	0.99 (0.97–1.02)	0.63		
MAX PV diameter, mm	1.141.0–1.31)	0.06	0.95 (0.56–1.62)	0.86
MIN PV diameter, mm	0.97 (0.83–1.15)	0.75		
EVL/EIS history	2.5 (0.89–6.99)	0.08	2.20 (0.49–9.91)	0.30
PV length, mm	0.96 (0.92–1.01)	0.12		
Angle of PV	0.94 (0.90–0.98)	0.005	0.96 (0.89–1.04)	0.34
Curvature of PV		0.007		0.12
Area of PV	1.01 (1.0–1.01)	0.07	1.02 (0.96–1.08)	0.53
Circumference of PV	1.03 (0.99–1.08)	0.13		
Hydraulic diameter of PV	1.14 (0.99–1.31)	0.07	0.73 (0.17–3.19)	0.67

EVL, endoscopic variceal ligation; EIS, endoscopic injection sclerotherapy

### CFD simulation

**Fig. 4:**
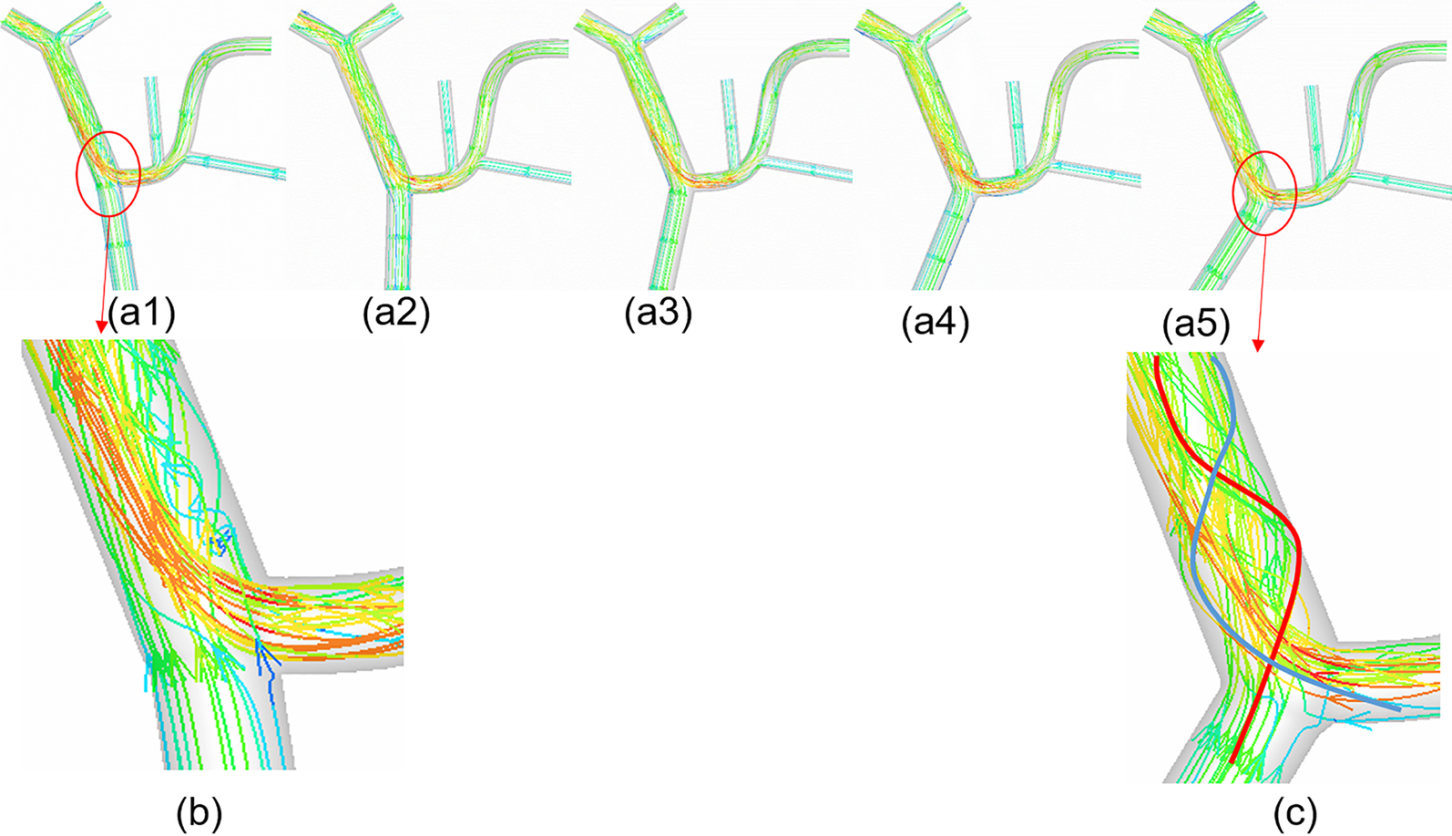
The flow patterns in the models; **a**_**1**_, **a**_**2**_, **a**_**3**_, **a**_**4**_, **a**_**5**_ represent the model with α = 80°, 90°, 100°, 110°, 120°, respectively; **b** and **c** show the flow detail at the local proximal portal vein in the ideal model with α = 80° and the model with α = 120°

**Fig. 5:**
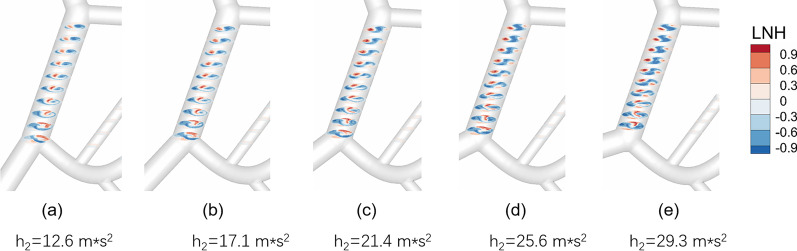
The effect of the angulation of SMV and SV on helical flow in the PV; **a**, **b**, **c**, **d**, **e** represent the model with α = 80°, 90°, 100°, 110°, 120°, respectively; h_2_, helical intensity; LNH, localized normalized helicity

Figures 4 and 5 show that with the change in angle α(the angulation of the SMV and SV), the flow pattern of blood flow changed greatly, especially the helical flow. When α = 80°, helical flow only appeared at the local PV near the intersection of SMV and SV (Fig. 5a). In addition, there were few spirals, and the main blood flowed smoothly in the PV (Fig. 4b); when α = 120°, most regions were occupied with helical flow (Fig. 5e), and distinct spirals were observed at the intersection which was composed of two blood flows from the SMV and SV (Fig. 4c). Specifically, the h_2_ gradually increased with increasing α. When *α* = 80°, *h*_2_ = 12.6 m/s^2^; when *α* = 120°, *h*_2_ = 29.3 m/s^2^, with increase of 133%. (Additional file 1: Table S1). In addition, the area-average WSS (AWSS) in the PV wall increased as the *α* increased. When *α* = 80°, AWSS = 1.61 Pa; when *α* = 120°, AWSS = 1.79 Pa, with an increase of 11%. However, the PV V_MAX_ and V_MEAN_ were almost unchanged when α varied from 80° to 120°, and the values remained to be close to 0.45 cm/s (0.44–0.46 cm/s) and 0.23 cm/s, respectively. In addition, the flow rate of LPV (or RPV) and the PV pressure also had only marginal changes when the angle α increased (Additional file 1: Table S1).

## Discussion

Early diagnosis and treatment of PVT can prevent the development of thrombus and the occurrence (or aggravation) of portal hypertension. This study attempted to determine the relationship between the morphological features and the occurrence or development of PVT. The results showed a positive relationship between the angulation of SMV and SV, and PVT incidence in cirrhotic patients that the angulation of the SMV and SV in patients with PVT was smaller than that in the patients without PVT (*p* < 0.0001). In addition, the numerical simulation of the idealized PV models suggested a fresh mechanism that the presence of helical flow may reduce the risk of PVT development.

The angulation of the SMV and SV was found to be the only independent risk factor for the presence of PVT in this study. Few studies have reported the association between morphological parameters and PVT, the diameters of PV and SV were suggested to be the risk factors for PVT development [19, 20]. Child–Pugh class B and C, nonselective beta blockers taking history, EVL/EIS treatment, splenectomy and presence of high-risk varices were risk factors for PVT in cirrhosis [21]. The above previously reported risk factors were not different between patients with PVT or without PVT subgroups in this study, except the history of EVL/EIS. We speculated that results may be decided by the patients included who are preparing for TIPS with the most end stage of liver cirrhosis.

In addition, the special structure, the splenomesenteric confluence (SMC) was found to promote helical flow patterns in the PV [16]. Helical flow has been proven to suppress flow disturbances and therefore is biologically beneficial. Preliminary studies demonstrated the widely existing helical flow might play positive physiological roles in facilitating blood flow transport, suppressing disturbed blood flow, preventing the accumulation of atherogenic low density lipoproteins on the luminal surfaces of arteries, enhancing oxygen transport from the blood to the arterial wall and reducing the adhesion of blood cells on the arterial surface [15]. Helical flow may have a physiological role in venous circulation, and its absence of it may be a feature of venous disease [22]. In this study, as the angulation of the SV and SMV increased, a significant helical flow appeared in the portal vein. This may be because the fact that the blood flow in the portal vein mainly comes from the SV and SMV; they confluence and interaction at the entrance of the portal veins and flow to the liver in a helical flow pattern, indicating that the varying angulation of the SV and SMV may change the portal flow hemodynamics to affect the development of the PVT and that the smaller angulation of the SV and SMV causes smaller helical flow with a greater possibility of PVT. Previous studies reported that portal vein (PV) velocity decreases to 15 cm/s, variceal bleeding and low platelets count were significant risk factors for PVT development [5]. Therefore, we can guess that the end-stage cirrhosis patient with portal vein (PV) velocity lower than 15 cm/s to measure the angulation of SMV and SV may acquire benefit to evaluate the risk of PVT development.

There are some limitations in the study. First, the number of the patients was small, and more cohorts are needed to verify our findings before they can be used in clinic. Second, previous studies reported that a portal vein velocity decrease to 15 cm/s can increase the risk of PVT development; therefore, we did not evaluate the effect of portal vein velocity on PVT development. Third, we did not include validation cohorts to validate our findings, and patients with the end-stage cirrhosis with regular follow-up are recommended for evaluation in further studies.

## Conclusion

In conclusion, the angulation of the SV and SMV was closely related to the formation of PVT. Numerical simulation analysis found that helical flow may change following the varying angulation of the SV and SMV. Therefore, angulation of the SV and SMV may help to earlier identify high-risk cohorts for future PVT earlier.

## Supplementary Information

**Additional file 1.** The hemodynamic parameters in the PV with different angulation of SMV and SV (Table S1), the Illustration of the ideal portal system (Figure S1), the ROC of angulation of SMV and SV for predicting PVT (Figure S2).

## Data Availability

The data are available for scrutiny from external requests.

## Abbreviations

ALT: Alanine aminotransferase
AST: Aspartate aminotransferase
EIS: Endoscopic injection sclerotherapy
EVL: Endoscopic variceal ligation
HBV: Hepatitis B virus
HCV: Hepatitis B virus
INR: International normalized ratio
LPV: Left portal vein
MELD: Model for end-stage liver disease
PLT: Platelet count
PPG: Portal pressure gradient
PT: Prothrombin time
PV: Portal vein
PVT: Portal vein thrombosis
RPV: Right portal vein
SMV: Superior mesenteric vein
SV: Splenic vein
