# Supplementary material for: A novel potential mechanism for the development of portal vein thrombosis in cirrhosis based on portal hemodynamics
Source: Insights Imaging. 2022 Dec 13;13:192. doi: 10.1186/s13244-022-01330-4 (PMC9748017; doi:10.1186/s13244-022-01330-4)
Supplement: Supplementary file 1 — Additional file 1. The hemodynamic parameters in the PV with different angulation of SMV and SV (Table S1), the Illustration of the ideal portal system (Figure S1), the ROC of angulation of SMV and SV for predicting PVT (Figure S2). [file 13244_2022_1330_MOESM1_ESM.pdf]

ELECTRONIC SUPPLEMENTARY MATERIAL

A novel potential mechanism for the development of portal vein thrombosis in cirrhosis based on portal hemodynamics

Table S1. The hemodynamic parameters in the PV.

α	h_2 （m/s^2）	V_{MEAN} (m/s)	V_{MAX} (m/s)	P (mmHg)	FR_{RPV} (ml/s)	FR_{LPV} (ml/s)	AWSS (pa)
α=80°	12.6	0.23	0.46	10.25	14.38	6.62	1.61
α=90°	17.1	0.23	0.45	10.24	14.46	6.53	1.64
α=100°	21.4	0.23	0.44	10.22	14.29	6.68	1.74
α=110°	25.6	0.23	0.44	10.25	14.21	6.77	1.76
α=120°	29.3	0.23	0.45	10.24	14.33	6.76	1.79

α, the angle of SMV and SV; h_2, helical strength; V_{MEAN}, average blood velocity; V_{MAX}, maximum blood velocity; P, pressure; FR_{LPV}, LPV flow rate; FR_{RPV}, RPV flow rate; AWSS, area-average wall shear stress.

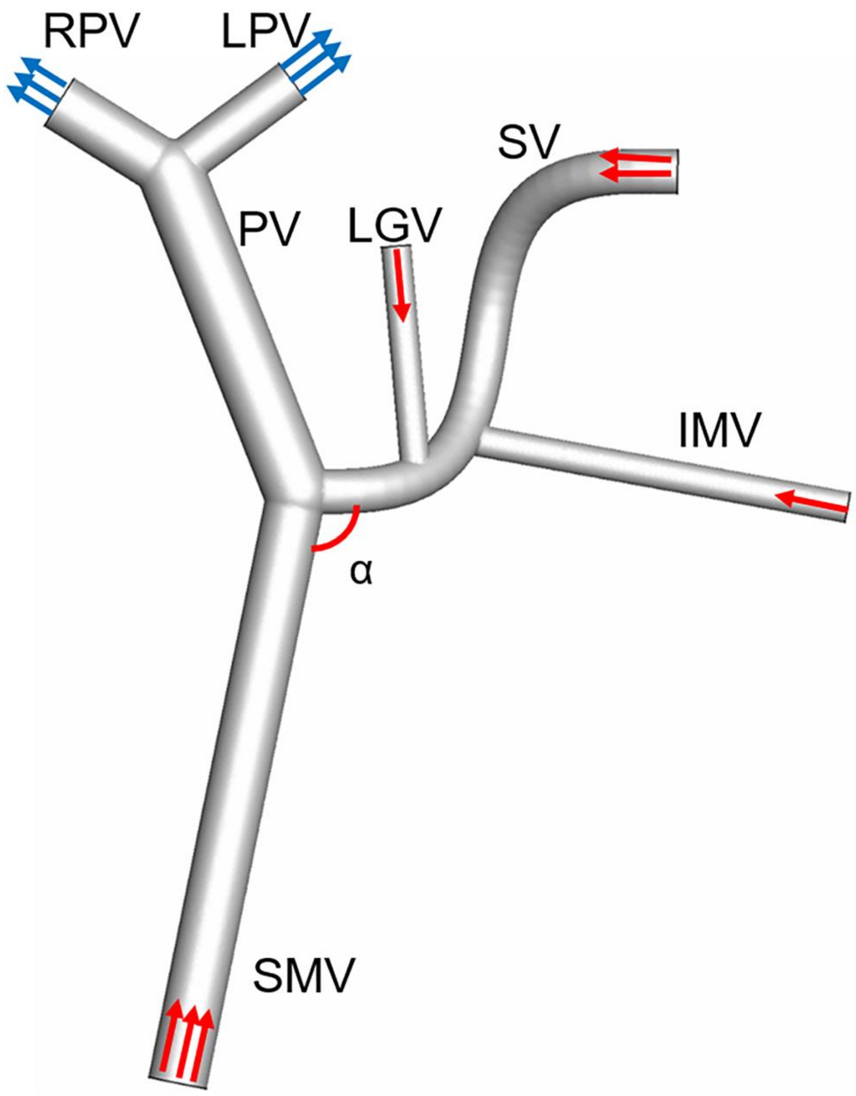

Figure S1. Illustration of the ideal portal system; LGV, left gastric vein; IMV, inferior mesenteric vein.

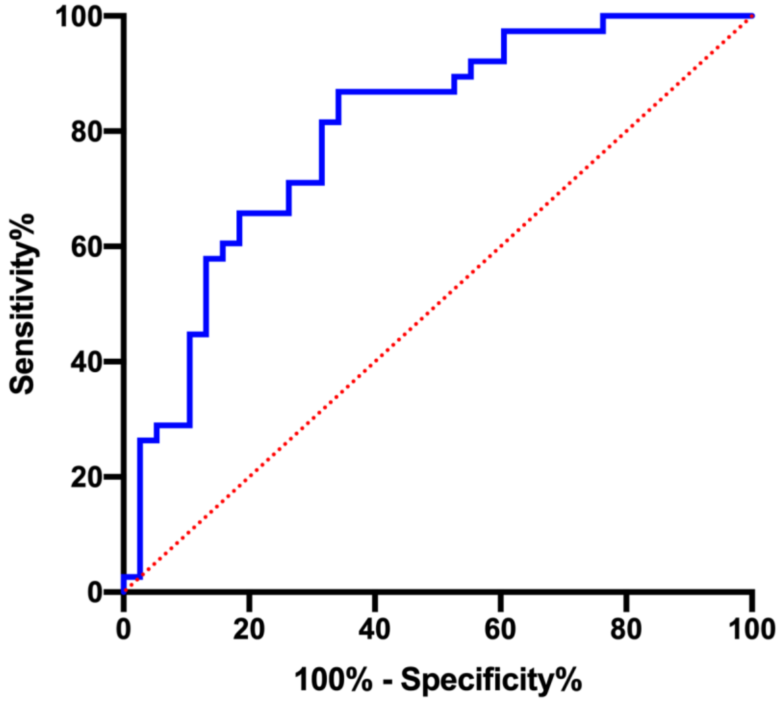

Figure S2. The receiving operating characteristic curve of angulation of SMV and SV for predicting PVT.
